# Sequencing, *de novo* assembling, and annotating the genome of the endangered Chinese crocodile lizard *Shinisaurus crocodilurus*

**DOI:** 10.1093/gigascience/gix041

**Published:** 2017-06-08

**Authors:** Jian Gao, Qiye Li, Zongji Wang, Yang Zhou, Paolo Martelli, Fang Li, Zijun Xiong, Jian Wang, Huanming Yang, Guojie Zhang

**Affiliations:** 1 BGI Education Center, University of Chinese Academy of Sciences, Shenzhen 518083, China; 2 China National Genebank, BGI-Shenzhen, Shenzhen 518083, China; 3 BGI-Shenzhen, Shenzhen 518083, China; 4 State Key Laboratory of Genetic Resources and Evolution, Kunming Institute of Zoology, Chinese Academy of Sciences, Kunming 650223, China; 5 Department of Molecular Evolution and Development, Faculty of Life Sciences, University of Vienna, Althanstrasse 14, 1090 Vienna, Austria; 6 Veterinary Department, Ocean Park Hong Kong, Hong Kong SAR; 7 James D. Watson Institute of Genome Sciences, Hangzhou 310058, China; 8 Centre for Social Evolution, Department of Biology, University of Copenhagen, Universitetsparken 15, DK-2100 Copenhagen, Denmark

**Keywords:** Chinese crocodile lizard, *Shinisaurus crocodilurus*, sequencing, genome assembly, annotation

## Abstract

The Chinese crocodile lizard, *Shinisaurus crocodilurus*, is the only living representative of the monotypic family Shinisauridae under the order Squamata. It is an obligate semi-aquatic, viviparous, diurnal species restricted to specific portions of mountainous locations in southwestern China and northeastern Vietnam. However, in the past several decades, this species has undergone a rapid decrease in population size due to illegal poaching and habitat disruption, making this unique reptile species endangered and listed in the Convention on International Trade in Endangered Species of Wild Fauna and Flora Appendix II since 1990. A proposal to uplist it to Appendix I was passed at the Convention on International Trade in Endangered Species of Wild Fauna and Flora Seventeenth meeting of the Conference of the Parties in 2016. To promote the conservation of this species, we sequenced the genome of a male Chinese crocodile lizard using a whole-genome shotgun strategy on the Illumina HiSeq 2000 platform. In total, we generated ∼291 Gb of raw sequencing data (×149 depth) from 13 libraries with insert sizes ranging from 250 bp to 40 kb. After filtering for polymerase chain reaction–duplicated and low-quality reads, ∼137 Gb of clean data (×70 depth) were obtained for genome assembly. We yielded a draft genome assembly with a total length of 2.24 Gb and an N50 scaffold size of 1.47 Mb. The assembled genome was predicted to contain 20 150 protein-coding genes and up to 1114 Mb (49.6%) of repetitive elements. The genomic resource of the Chinese crocodile lizard will contribute to deciphering the biology of this organism and provides an essential tool for conservation efforts. It also provides a valuable resource for future study of squamate evolution.

## Data Description

### Background

**Figure 1:**
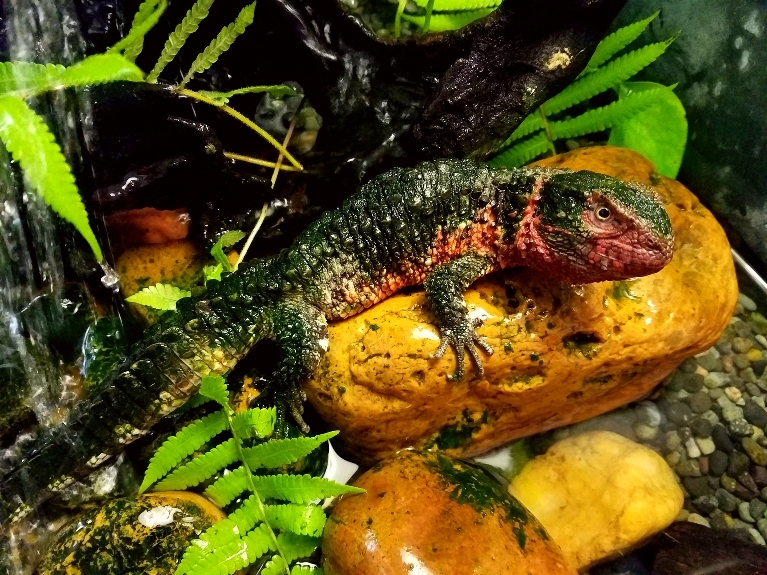
Example of a Chinese crocodile lizard, *Shinisaurus crocodilurus* (image from Wong Sai Lok).

The Chinese crocodile lizard, *Shinisaurus crocodilurus* (NCBI taxonomy ID 52224) (Fig. 1), was first collected in 1928. In 1930, to accommodate the monotypic genus and species, Ahl established Shinisauridae as a new family under the order Squamata [1]. The species usually is found along slow-flowing rocky streams in montane evergreen forests [2] and is distributed in the east part of the Guangxi Zhuang Autonomous Region, the west and north parts of Guangdong Province in southern China, and in mountainous areas of northern Vietnam [3]. It is a semi-aquatic diurnal predator and a strong swimmer, preying on fish, tadpoles, and aquatic insects. It is ovoviviparous and breeds in July and August in the wild [1, 4, 5]. A variety of anthropogenic hazards have caused severe population declines within the last decades. Illegal poaching for the international pet trade, traditional medicine, and food represents the main driver fueling the ongoing population decline [2]. A quantitative survey on the species was carried out in 1978, which estimated the total known population at 6000 individuals, and in 2008 it estimated a total population of 950 animals [5, 6]. The species was listed in the Convention on International Trade in Endangered Species of Wild Fauna and Flora (CITES) Appendix II in 1990 [7] and in the IUCN Red List of Threatened Species in 2014 [6], and a proposal to uplist it to Appendix I was passed at the CITES Seventeenth meeting of the Conference of the Parties in 2016 [8].

**Table 1:** Statistics of the Chinese crocodile lizard genome sequencing

			Raw data			Clean data		
Insert size (bp)	Library	Reads length (bp)	Total data (Gb)	Sequence coverage (×)	Physical coverage (×)	Total Data (Gb)	Sequence coverage (×)	Physical coverage (×)
250	1	150	54.16	27.78	23.15	41.99	23.15	20.32
500	1	150	54.67	28.04	46.73	39.27	46.73	39.5
S800	1	150	15.68	8.04	21.45	11.82	21.45	18.31
2000	2	49	34.93	17.92	365.62	13.99	7.18	146.48
5000	2	49	34.35	17.62	899.1	13.60	6.97	355.83
10 000	2	49	33.74	17.3	1765.7	9.48	4.86	496.14
20 000	2	49	30.64	15.71	3207.13	3.25	1.66	340.01
40 000	2	49	32.68	16.76	6842.73	3.33	1.71	697.58
Total	13	-	290.85	149.17	13 171.61	136.73	70.12	2114.17

Coverage calculation was based on the estimated genome size of 1.95 Gb. Sequence coverage is the average number of times a base is read, while physical coverage is the average number of times a base is spanned by mate-paired reads.

### Sample collection and sequencing

The genomic DNA of the Chinese crocodile lizard was extracted from the blood collected from the tail vein of a single adult male lizard in Ocean Park Hong Kong, which is a zoological theme park in Hong Kong. The venipuncture procedure was identical to that used for routine clinical blood draws in lizards in compliance with the Animal Welfare and Use Guidelines of Ocean Park. This lizard was alive in the collection of Ocean Park Hong Kong at the time of manuscript submission (Animal ID: MIG12–30 061 867; the CITES license to possess number is APO/PL 266/12). Three standard DNA libraries with short-insert sizes (250, 500, and 800 bp) and 10 mate-paired libraries with long-insert sizes (2 kb × 2, 5 kb × 2, 10 kb × 2, 20 kb × 2, and 40 kb × 2) were constructed with the standard protocol provided by Illumina (San Diego, CA, USA). Paired-end sequencing was performed for all the 13 libraries on the HiSeq 2000 platform according to the manufacturer's instructions (Illumina, San Diego, CA, USA). The sequenced read length was 150 bp for the short-insert libraries and 49 bp for the long-insert libraries. In total, about 290.85 Gb (×149) of raw reads were eventually produced (Table 1).

### Read filtering and genome size estimation

**Figure 2:**
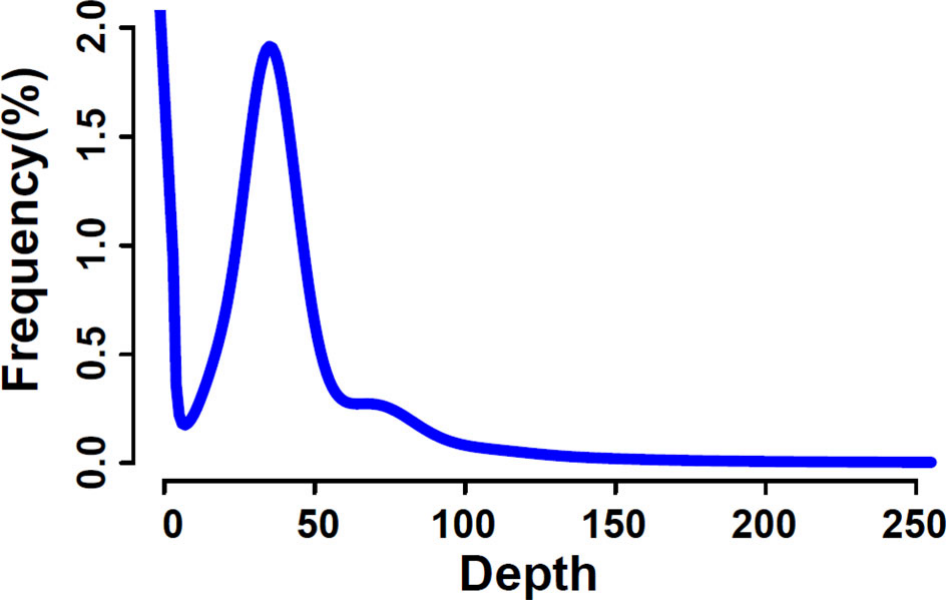
17-mer depth distribution. The 17-mer analysis was employed by using 250, 500, and 800 bp short-insert size libraries. The peak depth was ×35. The total number of 17-mer present in this subset was 68 234 898 814. The genome size was estimated to be 1.95 Gb according to the following formula: genome size = (Kmer number)/(Peak depth).

We obtained 136.73 Gb of clean data from the raw data by removing duplicated reads arising from polymerase chain reaction amplification during library construction, adapter-contaminated reads with ≥10 bp aligned to adapter sequence, low-quality reads that contain >5% “Ns” for the short-insert (250, 500, and 800 bp) data or >20% for the long-insert (2, 5, 10, 2, and 40 kb) data, and low-quality reads that contain ≥40 low-quality (Illumina phred quality score ≤ 7) bases for the short-insert data or ≥30 bases for the long-insert data using SOAPfilter, an application included in the SOAPdenovo package (SOAPdenovo2, RRID:SCR_014986) (Table 1) [9]. We obtained about 136.73 Gb of clean reads from about 290.85 Gb of raw reads; the total number of clean reads is 1 494 896 603, and the total number of raw reads is 3631.968,900. Then we used the clean data from the 3 short-insert (250, 500, and 800 bp) libraries to estimate the genome size of Chinese crocodile lizard with a 17-mer analysis [10]. A k-mer is related to an artificial sequence division of K nucleotides iteratively from sequencing reads [11]. We selected a fragment length of 17; the fragment is called 17-mer. When a certain coverage was reached, k-mer frequencies were plotted against the sequence depth gradient following a Poisson distribution [12], then the genome length could be estimated from the number and depth of kmer by the following formula: genome size = (Kmer number)/(Peak depth). According to that prediction, the Chinese crocodile lizard genome is estimated to be 1.95 Gb in size (Table 2; Fig. 2).

**Table 2:** Statistics of 17-mer analysis

Genome	Kmer	Kmer number	Peak depth	Estimated genome size (bp)	Used base (bp)
*Shinisaurus crocodilurus*	17	68 234 898 814	35	1 949 568 537	78 251 030 750

The genome size was estimated according to the following formula: genome size = (Kmer number)/(Peak depth).

### Genome assembly and completeness estimation

We employed the SOAPdenovo package (version 2.04.4) for genome assembly (SOAPdenovo2, RRID:SCR_014986) [9]. Briefly, the sequences derived from the short-insert libraries were first decomposed into k-mers to construct the *de Bruijn* graph, which was simplified to allow connection of the k-mers into a contiguous sequence (contigs). We tested a series of kmer lengths ranging from 31 to 81 bp, and the 69-mer was finally selected to generate a contig assembly with the longest N50 value. We then aligned the paired-end reads from both the small- and large-insert libraries to the contigs, calculated the support for relationships between contigs, and constructed scaffolds using distance information from read pairs. We required at least 3 and 5 read pairs to form a reliable connection between 2 contigs for short-insert and large-insert data, respectively. Finally, Kgf (version 1.16) [9] and GapCloser (GapCloser, RRID:SCR_015026; version 1.10.1) [9] were employed to close intra-scaffold gaps using paired-end reads from the small-insert libraries. The end result was a genome assembly with a total length of 2.24 Gb, scaffold and contig N50s of 1470 kb and 11.7 kb, respectively, and unclosed gap regions representing 7.98% of the assembly, which is comparable to the previously published reptile genome assemblies (Table 3).

**Table 3:** Comparison of genome assembly and gene number for 15 reptiles with published genomes

Species	Common name	Sequencing platform	Sequence coverage (×)	Assembled genome size (Gb)	Contig N50 (kb)	Scaffold N50 (kb)	Gap ratio (%)	Gene number	Reference
*Alligator mississippiensis*	American alligator	NGS	156.0	2.17	7.0	509	2.09	23 323	[40]
*Alligator sinensis*	Chinese alligator	NGS	109.0	2.30	23.4	2188	3.17	22 200	[41]
*Anolis carolinensis*	Green anole lizard	Sanger	6.0	1.78	79.9	4033	4.49	17 472	[42]
*Chrysemys picta bellii*	Western painted turtle	Sanger + NGS	18.0	2.59	11.9	5212	7.64	21 796	[43]
*Chelonia mydas*	Green sea turtle	NGS	82.3	2.24	20.4	3778	4.33	19 633	[44]
*Crocodylus porosus*	Saltwater crocodile	NGS	74.0	2.12	32.8	205	5.30	13 321	[40]
*Deinagkistrodon acutus*	Five-pacer viper	NGS	114.2	1.47	22.4	2122	5.29	21 194	[45]
*Eublepharis macularius*	Leopard gecko	NGS	135.8	2.02	20.0	664	1.76	24 755	[26]
*Gavialis gangeticus*	Indian gharial	NGS	81.0	2.88	14.2	127	2.22	14 043	[40]
*Gekko japonicus*	Japanese gecko	NGS	131.3	2.55	21.1	685	3.54	22 487	[46]
*Ophiophagus hannah*	King cobra	NGS	28.0	1.66	4.0	226	13.5	18 579	[19]
*Pelodiscus sinensis*	Soft-shell turtle	NGS	105.6	2.21	21.9	3331	4.35	23 649	[44]
*Pogona vitticeps*	Australian dragon lizard	NGS	179.1	1.82	31.3	2290	3.78	19 406	[20]
*Python molurus bivittatus*	Burmese python	NGS	20.0	1.44	10.7	208	3.52	25 385	[18]
*Shinisaurus crocodilurus*	Chinese crocodile lizard	NGS	149	2.24	11.7	1470	7.98	20 150	

We then employed Benchmarking Universal Single-Copy Orthologs (BUSCO; version 3.0.0) to evaluate the completeness of the assembly using 2586 vertebrata expected genes (BUSCO, RRID:SCR_015008) [13]. This analysis showed that 2391 (92.5%) and 125 (4.8%) of the 2586 expected vertebrata genes were identified as complete and fragmented, respectively, while 70 (2.7%) genes were considered missing in the assembly. We ran the same version of BUSCO to the other 14 retile genomes, respectively; the completeness of the Chinese crocodile lizard assembly was also comparable to other published reptile genome assemblies (Table 4).

**Table 4:** The percentages of complete, fragmented, and missing genes out of the 2586 expected vertebrata genes in 15 reptile genomes based on the BUSCO assessment

Species	Common name	Complete single-copy (%)	Complete duplicated (%)	Fragmented (%)	Missing (%)
*Alligator mississippiensis*	American alligator	95.0	0.6	3.1	1.3
*Alligator sinensis*	Chinese alligator	94.4	0.7	3.2	1.7
*Anolis carolinensis*	Green anole lizard	88.1	0.8	5.6	5.5
*Chelonia mydas*	Green sea turtle	93.9	0.8	3.7	1.6
*Chrysemys picta bellii*	Western painted turtle	75.5	0.8	3.3	20.4
*Crocodylus porosus*	Saltwater crocodile	94.1	0.6	2.1	3.2
*Deinagkistrodon acutus*	Five-pacer viper	94.5	0.6	2.4	2.5
*Eublepharis macularius*	Leopard gecko	94.0	1.2	3.3	1.5
*Gavialis gangeticus*	Indian gharial	85.2	0.5	11.6	2.7
*Gekko japonicus*	Japanese gecko	89.8	1.1	6.3	2.8
*Ophiophagus hannah*	King cobra	86.6	0.6	8.6	4.2
*Pelodiscus sinensis*	Soft-shell turtle	93.5	0.5	3.8	2.2
*Pogona vitticeps*	Australian dragon lizard	94.3	0.6	3.1	2.0
*Python molurus bivittatus*	Burmese python	91.0	0.7	5.4	2.9
*Shinisaurus crocodilurus*	Chinese crocodile lizard	91.6	0.9	4.8	2.7

### Repeat annotation

Repetitive elements in the Chinese crocodile lizard genome were identified by homology searches against known repeat databases and *de novo* predictions. Briefly, we identified known transposable elements (TEs) by using RepeatMasker (version 3.3.0; RepeatMasker, RRID:SCR_012954) [14] to search against the Repbase TE library (RepBase16.10) [14] and RepeatProteinMask within the RepeatMasker package to search against the TE protein database. We then employed RepeatModeler (version 1.0.5; RepeatModeler, RRID:SCR_015027) [15] and LTR_FINDER (version 1.0.5) [16] for *de novo* prediction of TEs. RepeatModeler was first used to construct a *de novo* crocodile lizard repeat library, which was subsequently used by RepeatMasker to annotate repeats in the crocodile lizard genome. LTR_FINDER was used to search the whole genome for a characteristic structure of the full-length long terminal repeat retrotransposons (LTRs), mainly based on their ∼18 bp terminal sequences being complementary to the 3’ tail of some tRNAs [16]. We provided LTR_FINDER with all eukaryotic tRNAs to search for LTRs. Finally, we searched the genome for tandem repeats using Tandem Repeats Finder (TRF; version 4.04) [17]. The results from different methods were presented in Table 5. Overall, a total of 1114 Mb of non-redundant repetitive sequences were identified, accounting for 49.6% of the Chinese crocodile lizard genome (Table 5), and by using RepeatMasker, we found that long interspersed elements are the most predominant elements in *de novo* predictions, which accounted for 10% of the genome. This lizard genome, in terms of repeat content, is similar to the known genomes of the Burmese python [18], king cobra [18, 19], Australian dragon lizard [20], and green anole lizard (Table 6) [21].

**Table 5:** The statistics of repeats annotated by different methods in the Chinese crocodile lizard genome

Method	Total repeat length (bp)	Percentage of genome
TRF	35 995 906	1.74
Repeatmasker	199 442 776	9.65
Proteinmask	164 914 070	7.98
RepeatModeler	938 017 292	41.79
LTR_FINDER	235 204 092	10.48
Total	1 113 900 339	49.62

**Table 6:** Breakdown of repeat content for 5 reptile genomes estimated by RepeatMasker

Repeat type	The Burmese python (%)	The king cobra (%)	The green anole lizard (%)	The Australian dragon lizard (%)	The Chinese crocodile lizard (%)
DNA	3.45	3.49	8.71	3.26	3.80
LINE	8.57	10.55	12.19	10.93	10.20
SINE	1.60	2.09	5.11	3.14	2.72
LTR	0.85	1.75	2.94	0.92	1.52
Unknown	12.61	12.87	7.49	16.23	23.95
Total	31.82	35.22	33.82	35.93	41.79

### Gene prediction

We combined homology-based and *de novo* methods to build consensus gene models of the reference genome. In the homology-based method, protein sequences of *Anolis carolinensis*, *Gallus gallus*, and *Homo sapiens* derived from the Ensembl database (release-67; Ensembl, RRID:SCR_002344) were first mapped to the Chinese crocodile lizard genome using TBLASTN (version 2.2.23; TBLASTN, RRID:SCR_011822) [22] with an E-value cutoff of 1e-5, and the BLAST hits were linked into candidate gene loci with GenBlastA (version 1.0.4) [23]. Then the genomic sequences of the candidate loci together with 2 kb flanking sequences were extracted, and gene structures were determined by aligning the homologous proteins to these extracted genomic sequences using GeneWise (version 2.2; GeneWise, RRID:SCR_015054) [24]. In the *de novo* method, we randomly chose 1000 homology-based gene models with intact open reading frames and an aligning rate of 100% (i.e., query protein length/predicted protein length) to train Augustus (version 2.5.5) (Augustus: Gene Prediction, RRID:SCR_008417) [25] in order to obtain gene parameters appropriate to the Chinese crocodile lizard genome. Then we performed *de novo* prediction on the repeat-masked genome using Augustus with the obtained gene parameters. Finally, gene models from these 2 methods were combined into a non-redundant gene set of 20 150 genes in the Chinese crocodile lizard using a similar strategy as Xiong et al. (Table 3) [26].

### Gene function annotation

Gene names were assigned according to the best hit of the alignments to the SwissProt and TrEMBL databases (Uniprot release 2011–06; UniProt, RRID:SCR_002380) [27] using BLASTP (version 2.2.3). The motifs and domains of genes were determined by InterProScan (version 4.7; InterProScan, RRID:SCR_005829) [28] against the InterPro protein signature databases including ProDom (ProDom, RRID:SCR_006969) [29], PRINTS (PRINTS, RRID:SCR_003412) [30], Pfam (Pfam, RRID:SCR_004726) [31], SMART (SMART, RRID:SCR_005026) [32], PANTHER (PANTHER, RRID:SCR_004869) [33], and (PROSITE PROSITE, RRID:SCR_003457) [34]. Gene ontology (GO; GO, RRID:SCR_002811) terms for each gene were obtained from the corresponding InterPro entries [35], and 14 518 genes were assigned to 1 or more GO terms. All genes were aligned against KEGG proteins (release 58; KEGG, RRID:SCR_012773) [36], and the pathway in which the gene might be involved was derived from the matched genes in KEGG. Overall, we inferred 20 010 (99.31%) genes annotated from the results of the 4 databases (Table 7).

**Table 7:** Number and percentage of genes with functional annotation

	Number	Percentage (%)
SwissProt	18 817	93.38
TrEMBL	9675	48.01
InterPro	17 589	87.29
KEGG	15 791	78.37
GO	14 518	72.05
Combined	20 010	99.31

## Conclusion

Here we report the first annotated Chinese crocodile lizard genome sequence assembly. This research yielded a draft genome assembly with a total length of 2.24 Gb and an N50 scaffold size of 1.47 Mb. The assembled genome was predicted to contain 20 150 protein-coding genes and up to 1114 Mb (49.6%) of repetitive elements. The draft genome and annotation will provide valuable data for the captive breeding and aid research into the phylogeny and biological features such as ovoviviparity, venom glands, etc. [37, 38].

## Abbreviations

BUSCO: Benchmarking Universal Single-Copy Orthologs; CITES: the Convention on International Trade in Endangered Species of Wild Fauna and Flora; Gb: Gigabase; GO: gene ontology; LTRs: terminal repeat retrotransposons; TE: transposable element.

## Funding

This work was funded by the China National Genebank, and the Project U1301252 was supported by National Natural Science Foundation of China. The sample was supplied by the Genome 10K (G10K) consortium. We would like to thank the faculty and staff in BGI-Shenzhen, who contributed to the sequencing of the Chinese crocodile lizard genome.

## Availability of supporting data

Supporting data for this Data Note are available in the GigaScience database (GigaDB) [39]. Raw data are available in the SRA database with number SRA492579 and Bioproject accession PRJNA353147.

## Competing interests

The authors declare that they have no competing interests.

## Author contributions

## Supplementary Material

Reviewer_1_Report_(Original_Submission).pdf

Reviewer_2_Report_(Original_Submission).pdf
