# Supplementary material for: Sequencing, de novo assembling, and annotating the genome of the endangered Chinese crocodile lizard Shinisaurus crocodilurus
Source: Gigascience. 2017 Jun 8;6(7):1–6. doi: 10.1093/gigascience/gix041 (PMC5569961; doi:10.1093/gigascience/gix041)
Supplement: Reviewer_2_Report_(Original_Submission).pdf [file gix041_Reviewer_2_Report_(Original_Submission).pdf]

Reviewer Report

Title: Sequencing, de novo assembling and annotating the genome of the endangered Chinese crocodile lizard Shinisaurus crocodilurus

Version: Revision 2 **Date:** 12/12/2016

Reviewer name: Anthony Geneva

Reviewer Comments to Author:

The manuscript under consideration, "Sequencing, de novo assembling and annotating the genome of the endangered Chinese crocodile lizard Shinisaurus crocodilurus" presents the first genome sequence and annotation of this unique lizard species. The authors describe data collection and genome assembly for this species, as well as repeat, gene, and functional annotation. The methods used are sound and well described and this paper represents an important contribution to understanding the biology of this species and adds to the relatively small set of existing reptile genomes sequenced to date.

Despite the overall quality of the manuscript there are a few areas that require improvement. Currently the background information regarding the Chinese crocodile lizard and concluding comments are limited to the abstract. This information must also be included in the main document with greater detail. I suggest adding a "Background" heading under "Data Description". In this section, the background information provided in the abstract should be expanded upon, and relevant literature should be cited. A "Conclusion" section should be added to the end of the main manuscript, again expanding upon the information summarized in the Abstract. Please see the recent GigaScience paper "Draft Genome of the lined seahorse, Hippocamus erectus" for an appropriate example.

All analyses performed seem to be appropriate, but in the case of the BUSCO analyses the software version and underlying ortholog database used are out of data. More recent releases should be applied to the data. In addition, some analyses (indicated in the specific comments below) should be described in more detail for readers.

Finally, while the current form of this paper is written in a clear and straight-forward manner, the manuscript can be improved by a final round of proofreading for clarity and grammar. I have provided some suggestions to improve readability below, but these represent the most difficult passages and do not represent all places where clarity could be improved.

Specific Comments
Please note the review copy of the manuscript included two sets of line numbers. All comments below refer to the inner set of line numbers.

Abstract: The "Background" and "Conclusion" sections of the abstract contain information that appears nowhere in the main manuscript. This needs to be corrected. I suggest adding these sections in greater

detail and with supporting citations to the main text.

Line 27-29: When added to the main manuscript please add citations supporting claims of species distribution and the causes and magnitude of reduced population size.

Line 42: When added to the main manuscript the authors should provide detail about how the reported genome will be useful to deciphering the biology of the Chinese crocodile lizard and what it will contribute to conservation efforts.

Line 41: I suggest the authors change "would" to "will"

Line 43: I suggest the authors change "study of squamata evolution" to either "study of squamate evolution" or "study of the evolution of the squamata"

Line 54: Please describe what "Ocean Park Hong Kong" is. Is this a zoo, research facility, or something else?

Line 56-57: I suggest the authors change "This lizard is still alive in the collection of Ocean Park Hong Kong when the manuscript is finished" to "This lizard was alive in the collection of Ocean Park Hong Kong when at the time of manuscript submission."

Line 61: Please provide manufacturer details, citations, and/or methods for all libraries that were prepared. For example, did you use TrueSeq, Nextera, or some other approach? Did you make any modifications to these protocols?

Line 76: Please report coverage and number of reads before and after quality filtering.

Line 78: Please briefly describe this 17-mer analysis and how it generates an estimate of genome size.

Line 82: Why is citation [2] (the Giant Panda genome paper) cited here?

Line 86: What were the k-mer lengths considered?

Line 97: This analysis should be redone with the most recent BUSCO release (v3) which incorporates a much large database of orthologs (OrthoDB9) and has a prebuilt database for tetrapods (instead of the broader vertebrate category used here)

Line 119-123: Why do the totals between Table 5 and 6 differ? Shouldn't the totals be the same, just divided among detection method (Table 5) and repeat class (Table 6)?

Line 149: Please provide citations for the databases used.

Line 154: I suggest the authors change "annotated" to "annotations"

Line 157: Is there a unique identifier associated with this record in GigaDB? If so, please provide it here.

Table 4: Did the authors perform these analyses across all species or just Shinisaurus? If so, this should be made clear in the text. If not, then sources for these results should be cited.

Level of Interest

Please indicate how interesting you found the manuscript: An article whose findings are important to those with closely related research interests

Quality of Written English

Please indicate the quality of language in the manuscript: Needs some language corrections before being published

Declaration of Competing Interests

Please complete a declaration of competing interests, considering the following questions:

- Have you in the past five years received reimbursements, fees, funding, or salary from an organisation that may in any way gain or lose financially from the publication of this manuscript, either now or in the future?
- Do you hold any stocks or shares in an organisation that may in any way gain or lose financially from the publication of this manuscript, either now or in the future?
- Do you hold or are you currently applying for any patents relating to the content of the manuscript?
- Have you received reimbursements, fees, funding, or salary from an organization that holds or has applied for patents relating to the content of the manuscript?
- Do you have any other financial competing interests?
- Do you have any non-financial competing interests in relation to this paper?

If you can answer no to all of the above, write 'I declare that I have no competing interests' below. If your reply is yes to any, please give details below.

I declare that I have no competing interests.

I agree to the open peer review policy of the journal. I understand that my name will be included on my report to the authors and, if the manuscript is accepted for publication, my named report including any attachments I upload will be posted on the website along with the authors' responses. I agree for my report to be made available under an Open Access Creative Commons CC-BY license (http://creativecommons.org/licenses/by/4.0/). I understand that any comments which I do not wish to be included in my named report can be included as confidential comments to the editors, which will not be published.

I agree to the open peer review policy of the journal
